# Amelioration of 6-OHDA-Induced Parkinson’s Symptoms in Zebrafish Larvae by an Almond Skin Acetonic Extract

**DOI:** 10.3390/ijms27062590

**Published:** 2026-03-12

**Authors:** Patrícia Carneiro, Patricia Pais, Ivo Vaz Oliveira, Sandra M. Monteiro, Carlos Venâncio, Luís Félix

**Affiliations:** 1 School of Life and Environmental Sciences (ECVA), University of Trás-os-Montes and Alto Douro (UTAD), 5000-801 Vila Real, Portugal; 2 Centre for the Research and Technology of Agro-Environmental and Biological Sciences (CITAB), Institute for Innovation, Capacity Building and Sustainability of Agri-Food Production (Inov4Agro), University of Trás-os-Montes and Alto Douro (UTAD), 5000-801 Vila Real, Portugal; ivo.vaz.oliveira@utad.pt (I.V.O.); smonteir@utad.pt (S.M.M.); cvenanci@utad.pt (C.V.); 3 Animal and Veterinary Research Centre (CECAV), University of Trás-os-Montes and Alto Douro (UTAD), 5000-801 Vila Real, Portugal

**Keywords:** Parkinson’s disease, zebrafish, 6-OHDA, behaviour, oxidative stress, almond skin

## Abstract

Parkinson’s disease (PD) is a neurodegenerative disorder characterized by mitochondrial dysfunction, oxidative stress, and apoptosis. Natural products rich in polyphenols have been investigated for their potential to modulate pathways associated with PD-related pathology. The present study evaluated the effects of an acetonic almond skin extract, an agri-food by-product, in a zebrafish (*Danio rerio*) larval model of PD induced by 6-hydroxydopamine (6-OHDA). Embryos were exposed to 250 µM 6-OHDA alone or in combination with the extract (5 and 25 µg/mL) from 48 to 120 h post-fertilization (hpf). Developmental parameters, locomotor behaviour, oxidative stress biomarkers, apoptosis, mitochondrial membrane potential, and tyrosine hydroxylase (TH) immunoreactivity were assessed at 120 hpf. Exposure to 6-OHDA reduced TH immunofluorescence and impaired locomotor performance, accompanied by increased apoptotic signal and mild alterations in mitochondrial membrane potential. Co-exposure to the almond skin extract attenuated the reduction in TH immunoreactivity and partially modulated behavioural outcomes in a concentration-dependent manner. The extract alone increased glutathione S-transferase (GST) activity and reduced reactive oxygen species (ROS) levels, suggesting modulation of redox-related pathways. Notably, the highest concentration restored the TH signal but did not fully normalize the behavioural endpoints, indicating potential concentration-dependent complexity. Although sustained oxidative stress was not detected at the assessed time point, the observed mitochondrial and apoptotic alterations suggest involvement of multiple cellular processes. However, detailed mechanistic pathways were not directly investigated. Overall, these findings indicate that the almond skin extract modulates dopaminergic and behavioural alterations in a PD-induced zebrafish model, supporting its potential as a source of bioactive compounds, warranting further mechanistic and translational investigation.

## 1. Introduction

Parkinson’s disease (PD) is a progressive neurodegenerative disorder that primarily affects motor control due to the degeneration of dopaminergic neurons in the substantia nigra pars compacta [1]. Although the precise etiology of PD remains unclear, the disease is widely considered to arise from a multifactorial interplay of genetic and environmental factors, with mitochondrial dysfunction playing a critical role in disease onset and progression [2,3,4]. Beyond their fundamental role in adenosine triphosphate (ATP) production through oxidative phosphorylation, mitochondria are versatile and vital organelles involved in multiple cellular processes, such as apoptosis, calcium homeostasis, and the regulation of oxidative stress [5,6]. Impairment of mitochondrial function can lead to excessive production of reactive oxygen species (ROS), contributing to oxidative damage and neuronal vulnerability, key hallmarks of PD pathology [7].

In this context, natural products have attracted considerable attention as potential neuroprotective agents capable of modulating mitochondrial function and oxidative stress, thereby attenuating PD progression and symptom severity [8,9]. In particular, dietary compounds such as polyphenols, flavonoids, and alkaloids have been associated with neuroprotective effects in PD models, largely due to their antioxidant and anti-inflammatory properties [10,11,12]. However, the processing of natural products from the agri-food sector generates substantial quantities of waste biomass, which is often discarded despite representing an important source of high-value-added bioactive compounds [13,14]. The valorisation of agri-food by-products aligns with sustainable and circular economy principles, while simultaneously offering opportunities for the discovery of novel therapeutic agents. Recent studies have shown that the agri-food waste biomass is rich in secondary metabolites, such as polyphenols, flavonoids, and terpenoids [15]. Among these by-products, almond by-products, particularly almond skins, have gained increasing interest due to their high content of phenolic compounds and associated biological properties [16,17]. The high polyphenol content of almond skins [18] confers a strong antioxidant potential, which may be relevant for counteracting oxidative stress-driven neurodegenerative processes, such as those observed in PD [19]. These characteristics position almond skin extracts as promising candidates for the development of complementary or adjunctive therapeutic strategies for PD management. However, to date, the neuroprotective potential of almond skin extracts in the context of PD has not been explored.

To investigate the molecular and cellular mechanisms underlying PD, a wide range of experimental models have been developed, including toxin-induced and genetically modified in vivo systems (reviewed by Pingale et al. [20] and Thirugnanam et al. [21]). Among toxin-based PD models, 6-hydroxydopamine (6-OHDA) is widely used to induce dopaminergic impairment through selective catecholaminergic toxicity and mitochondrial dysfunction [22]. While in vitro approaches provide valuable mechanistic insights, they often fail to replicate the complex interactions and compensatory responses present in whole organisms [23]. Consequently, in vivo models remain essential for assessing integrated neurobiological and behavioural outcomes. In this context, zebrafish (*Danio rerio*) has emerged as an excellent model organism for PD research due to its conserved dopaminergic system, well-characterized neuroanatomy, and suitability for behavioural and molecular analyses [24,25,26]. Importantly, zebrafish models enable the simultaneous assessment of biochemical, cellular, and behavioural endpoints relevant to PD pathology. The present study aimed to evaluate, for the first time, the neuroprotective potential of almond skin extracts in a zebrafish model of 6-OHDA-induced PD. Specifically, the study assessed behavioural alterations, oxidative stress-related biomarkers, and dopaminergic neuron degeneration through immunostaining. By addressing multiple hallmarks of PD, this work provides novel insights into the potential application of agri-food by-products as sustainable sources of neuroprotective agents.

## 2. Results

### 2.1. Reduced Body Length in Larvae Exposed to 6-OHDA

**Figure 1:**
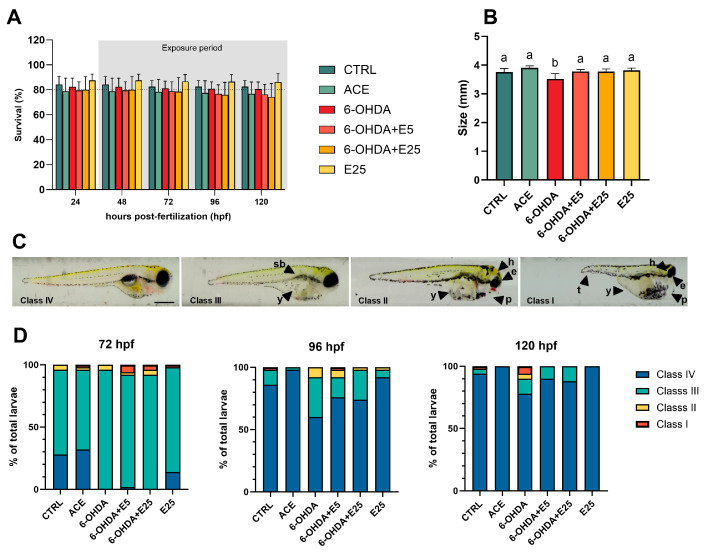
Morphological assessment of zebrafish larvae during the experimental period. (**A**) Cumulative mortality recorded throughout the exposure period. Data are presented as mean ± SD from five independent replicates. No statistically significant differences were observed between treatment groups. (**B**) Larval body length measurements. Values represent mean ± SD from five independent replicates (10 larvae per treatment per replicate, *n* = 1). Different letters indicate statistical differences among treatments (*p* < 0.05). (**C**) Representative images illustrating the morphological classes assigned based on treatment-induced alterations. Scale bar = 500 μm. sb: swimbladder; y: yolk sac; h: head; e: eye; p: pericardium; t: tail. (**D**) Distribution of larvae across morphological classes at 72, 96, and 120 hpf for each treatment group. Data are expressed as mean percentages (*n* = 5, with 10 larvae evaluated per replicate). CTRL (E3 medium), ACE (0.05% acetone), 6-OHDA (250 µM), 6-OHDA+E5 (250 µM 6-OHDA + 5 µg/mL extract), 6-OHDA+E25 (250 µM 6-OHDA + 25 µg/mL extract), and E25 (25 µg/mL extract).

Mortality was observed consistently throughout the experimental period, with detailed results presented in Figure 1A. Mortality in the control groups remained consistently below 20% and did not differ significantly among treatments at any evaluated time point (24 hpf: X^2^(5) = 4.835, *p* = 0.436; 48 hpf: X^2^(5) = 4.835, *p* = 0.436; 72 hpf: F(5, 24) = 0.875, *p* = 0.513; 96 hpf: X^2^(5) = 6.947, *p* = 0.225; and 120 hpf: X^2^(5) = 7.056, *p* = 0.217). Larval morphology was not significantly affected by the treatments, with comparable frequencies of developmental abnormalities observed across groups (*p* > 0.05). Minor Class I malformations (more severe) were sporadically detected in some treatments, with a higher incidence observed in the 6-OHDA-exposed group. Morphological scores assessed at later developmental stages (96 and 120 hpf) remained comparable among treatments (*p* > 0.05), despite the higher incidence of malformations in the group exposed to 6-OHDA. As shown in Figure 1B, exposure to 6-OHDA resulted in a significant reduction in embryos’ body size (F(5, 23) = 7.250, *p* = 0.0003) compared to both the control (*p* = 0.003), acetone (*p* < 0.0001), and co-exposed groups (*p* < 0.011). Exposure to the almond skin extract alone did not induce significant changes relative to the control group. In contrast, co-exposure with the extract significantly mitigated the reduction in body length induced by 6-OHDA (*p* = 0.002).

### 2.2. Restoration of Tyrosine Hydroxylase (TH)—Immunoreactivity in Dopaminergic Regions by the Almond Skin Extract

**Figure 2:**
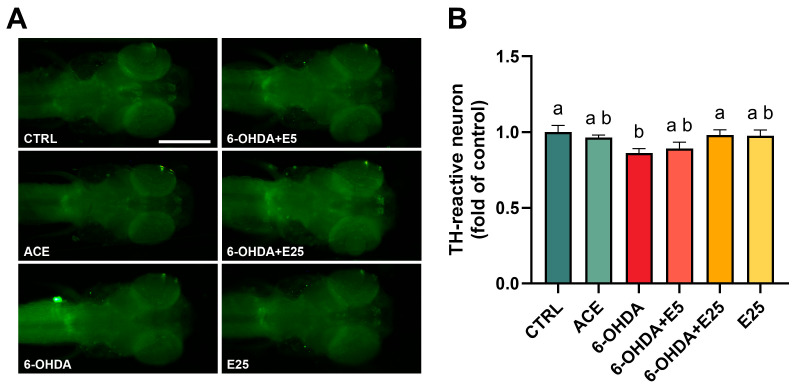
Tyrosine hydroxylase (TH) immunofluorescence at 120 hpf. (**A**) Representative whole-mount images of TH immunostaining in control, 6-OHDA, co-treatment, and extract-alone groups. Images were acquired under identical magnification, exposure time, illumination intensity, and gain settings. Scale bar = 500 µm. (**B**) Quantification of TH immunofluorescence intensity within anatomically defined regions of interest, expressed as fold of control. Data are presented as mean ± SD from five independent replicates (10 larvae per treatment per replicate, *n* = 1). Statistical comparisons were performed using the one-way ANOVA test followed by Tukey’s post hoc test. Different letters denote statistically significant differences among treatments (*p* < 0.05). CTRL (E3 medium), ACE (0.05% acetone), 6-OHDA (250 µM), 6-OHDA+E5 (250 µM 6-OHDA + 5 µg/mL extract), 6-OHDA+E25 (250 µM 6-OHDA + 25 µg/mL extract), and E25 (25 µg/mL extract).

At 120 hpf, TH-immunoreactive neurons were visualized, and the fluorescence quantification is shown in Figure 2 (X^2^(5) = 18.70, *p* = 0.002). Exposure to 6-OHDA resulted in a significant reduction (*p* = 0.014) in TH-associated fluorescence intensity in dopaminergic brain regions, compared to the control group. Co-exposure to the almond skin acetonic extract attenuated the 6-OHDA-induced reduction in TH fluorescence, with the highest extract concentration resulting in a significant increase relative to the 6-OHDA group (*p* = 0.045). Fluorescence levels in co-exposed larvae were comparable to those observed in both the control and extract-only groups.

### 2.3. Almond Skin Extract Improves Locomotor Performance

**Figure 3:**
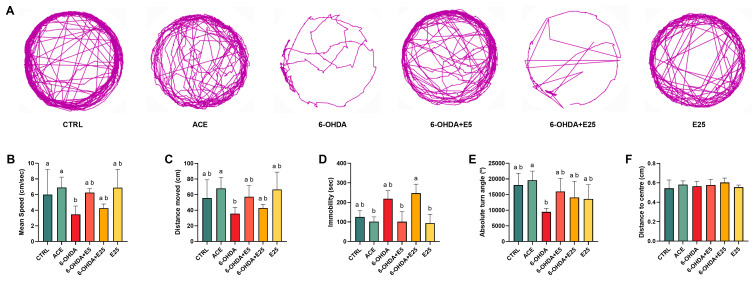
Locomotor activity analysis of zebrafish larvae at 120 hpf. (**A**) Representative locomotion tracking paths; (**B**) swimming speed; (**C**) total distance travelled; (**D**) immobility time; (**E**) turning angle; and (**F**) distance to centre. Data are presented as median values with interquartile ranges or as mean ± SD (**F**) based on 5 independent replicates (10 larvae per replicate, *n* = 1). Statistical analysis was conducted using the Kruskal–Wallis test, followed by Dunn’s test or one-way ANOVA test, followed by Tukey’s post hoc test. Different letters indicate statistically significant differences between treatment groups (*p* < 0.05). CTRL (E3 medium), ACE (0.05% acetone), 6-OHDA (250 µM), 6-OHDA+E5 (250 µM 6-OHDA + 5 µg/mL extract), 6-OHDA+E25 (250 µM 6-OHDA + 25 µg/mL extract), and E25 (25 µg/mL extract).

Behavioural outcomes assessed at 120 hpf are summarized in Figure 3. Representative swimming paths are shown in Figure 3A. Both average swimming speed (Figure 3B, X^2^(5) = 17.33, *p* = 0.004) and total distance moved (Figure 3C, X^2^(5) = 13.89, *p* = 0.016) were significantly affected by treatment. Larvae exposed to 6-OHDA exhibited a significant reduction in swimming speed compared to the control (*p* = 0.045) and acetone groups (*p* = 0.037). A significant reduction in total distance travelled was observed only when compared to the acetone group (*p* = 0.046). A non-significant trend toward increased distance moved was observed at the lowest concentration of the almond skin extract, with no significant differences relative to the control group (*p* > 0.05). Immobility time (Figure 3D, X^2^(5) = 19.27, *p* = 0.002) showed a non-significant increase following 6-OHDA exposure. Co-exposure with the highest almond skin extract concentration resulted in a significant increase in immobility compared to acetone, co-exposure with 5 µg/mL, and extract-only exposure at 25 µg/mL (*p* < 0.05). For mean absolute turn angle (Figure 3E, X^2^(5) = 11.21, *p* = 0.047), larvae exposed to 6-OHDA showed a significant decrease compared to the acetone group (*p* = 0.048). In contrast, no significant differences were detected relative to other treatments (*p* > 0.05). Concerning the distance to the centre of the well (F(5, 24) = 0.647, *p* = 0.666), no differences were observed among treatments.

### 2.4. Increased Apoptosis Induced by 6-OHDA Exposure

**Figure 4:**
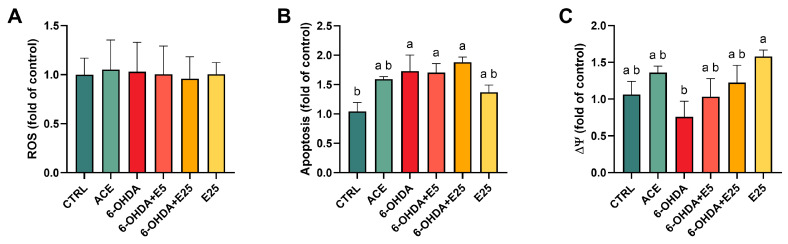
Assessment of oxidative stress and cell health markers in 120 hpf zebrafish larvae. (**A**) Reactive oxygen species (ROS) levels, (**B**) apoptosis, and (**C**) mitochondrial membrane potential. Data are normalized to control values and presented as median and interquartile ranges from five independent replicates (10 larvae per replicate, *n* = 1). Statistical analysis was performed using the Kruskal–Wallis test followed by Dunn’s test. Different letters indicate statistically significant differences between treatment groups (*p* < 0.05). CTRL (E3 medium), ACE (0.05% acetone), 6-OHDA (250 µM), 6-OHDA+E5 (250 µM 6-OHDA + 5 µg/mL extract), 6-OHDA+E25 (250 µM 6-OHDA + 25 µg/mL extract), and E25 (25 µg/mL extract).

At 120 hpf, ROS levels, apoptotic activity, and mitochondrial membrane potential were evaluated, using specialized fluorescent probes (Figure 4). No significant differences were observed in ROS levels among treatments (X^2^(5) = 1.495, *p* = 0.914). In contrast, exposure to 6-OHDA increased apoptotic levels (X^2^(5) = 19.22, *p* = 0.002) corresponding to a 1.75-fold increase relative to the control group (*p* = 0.043). Co-exposure with almond skin extract did not significantly reduce apoptosis compared to the 6-OHDA-treated group (*p* > 0.05). Exposure to acetone or the highest extract concentration alone did not significantly affect apoptosis compared to the control group (*p* > 0.05). Mitochondrial membrane potential differed significantly among treatments (X^2^(5) = 16.90, *p* = 0.005). Although 6-OHDA exposure induced a non-significant tendency toward ΔΨm reduction in comparison to the control group, increasing concentrations of the almond skin extract were associated with a tendency to normalize. Extract-only exposure caused an increase in the ΔΨm compared to 6-OHDA (*p* = 0.005).

### 2.5. Limited Biochemical Alterations Following Exposure

At 120 hpf, different biochemical parameters were assessed, and the results are summarized in Table 1. Although the statistical analysis yielded significant distributions, exposure to 6-OHDA did not result in significant alterations in the evaluated biochemical endpoints compared to controls (*p* > 0.05), with only subtle variations observed among 6-OHDA-treated groups. The most pronounced effect was observed for GST activity, which was significantly increased following exposure to the highest concentration of almond skin extract compared to the control group (*p* = 0.010).

## 3. Discussion

PD is characterized by dopaminergic dysfunction accompanied by mitochondrial alterations, oxidative imbalance, and neuronal vulnerability. These interconnected processes represent key therapeutic targets in experimental PD models. Natural products rich in polyphenols have attracted increasing attention due to their ability to modulate redox signalling and mitochondrial pathways. In this context, the present study investigated, for the first time, the effects of an acetonic almond skin extract in a 6-OHDA-induced zebrafish model, evaluating its impact on dopaminergic integrity, behavioural outcomes, and related biochemical parameters. Overall, the extract promoted preservation of TH immunoreactivity and normalized growth parameters, whereas behavioural and mitochondrial-related responses were concentration-dependent.

Despite the neurotoxic properties of 6-OHDA [27,28,29], exposure to this compound during zebrafish development did not result in increased mortality. This lack of lethality aligns with previous works using similar concentrations and exposure scenarios [30,31], although it contrasts with studies reporting higher toxicity at elevated doses [32]. These findings suggest the existence of a developmental and concentration-dependent threshold for 6-OHDA neurotoxicity, whereby survival is maintained despite potential cellular and functional impairments. Despite conflicting data regarding mortality outcomes, this study and others [30,33] consistently report an absence of gross morphological abnormalities in zebrafish exposed to 6-OHDA. This suggests that early developmental integrity remains unaffected under sublethal neurotoxic challenge. In accordance, Kesh et al. [31] reported mortality and developmental abnormalities only at higher concentrations of 6-OHDA (350 µM).

Exposure to 6-OHDA resulted in reduced larval body length without inducing gross morphological abnormalities, consistent with previous reports [30,34]. This pattern suggests that, at the tested concentration, 6-OHDA does not compromise overall developmental integrity but selectively interferes with growth-related processes. Similar reductions in body size have been associated with delayed skeletal development and impaired cellular proliferation [35,36]. Importantly, co-exposure to the almond skin extract restored body length, reinforcing the hypothesis that plant-derived compounds can mitigate neurotoxin-induced developmental impairments [37]. This protective effect may be attributed to the rich phytochemical composition of almond skins [38], as polyphenol-rich plant extracts have been shown to act as growth promoters, immunostimulants, and modulators of oxidative balance [39].

At the neuronal level, 6-OHDA exposure caused a significant reduction in the fluorescence intensity of TH-positive dopaminergic neurons. TH is a well-established marker for dopaminergic and noradrenergic neurons, representing the rate-limiting enzyme in dopamine synthesis [40]. The observed decrease in TH immunoreactivity suggests impairment of dopaminergic neuron integrity, leading to dopamine deficiency, a hallmark of PD [1]. This aligns with the extensive use of 6-OHDA in animal models to induce PD-like neurodegeneration, as this neurotoxin selectively targets dopaminergic neurons [41,42,43]. In zebrafish, similar reductions in TH-positive neurons following 6-OHDA exposure have been widely reported [30,44,45,46]. Reductions in dopaminergic function are typically accompanied by behavioural impairments, including reduced locomotor activity and altered motor coordination, which are hallmark features of PD-like phenotypes [47]. Accordingly, behavioural deficits were observed in the present study and are consistent with findings across different animal models [27,29,30,48,49,50,51,52]. Notably, co-exposure to the acetonic almond skin extract tended to decrease 6-OHDA-induced effects, restoring TH immunoreactivity to levels comparable to controls. While the lowest extract concentration appeared to confer partial behavioural protection, the highest concentration did not prevent behavioural deficits, despite suggesting preservation of TH signal and growth parameters. This dissociation suggests that behavioural endpoints may be more sensitive to concentration-dependent or systemic effects of the extract that are not directly reflected in dopaminergic immunoreactivity. It is possible that at higher concentrations the extract exerts additional neuromodulatory actions beyond dopaminergic pathways, potentially influencing locomotor behaviour through other neurotransmitter systems or mild sedative-like effects. The apparent discrepancy may be due to a potential concentration-dependent mechanism of the almond skin acetonic extract, despite the well-established beneficial effects of plant-based compounds in PD [53,54]. While this requires further scientific exploration as the effects of polyphenols are multifaceted [55], studies have shown that certain natural products can protect against zebrafish PD-induced effects through mechanisms involving the regulation of antioxidant signalling pathways [34,56,57,58,59].

The neurotoxic effects of 6-OHDA have been linked to the generation of oxidative radicals and inhibition of mitochondrial respiratory chain Complexes I and IV [60,61,62]. However, in the current study, no significant alterations in ROS production or oxidative stress biomarkers were observed. While this observation aligns with zebrafish studies under similar conditions [29,30], contradictory reports [56,57] indicate that oxidative outcomes may be sensitive to experimental design and exposure window. Despite the absence of overt oxidative stress, a decrease in mitochondrial membrane potential and a significant increase in apoptosis were observed. Although oxidative stress is classically associated with 6-OHDA toxicity, ROS and oxidative biomarkers were assessed only at the end of the exposure period (120 hpf). Therefore, transient early oxidative events may have occurred and resolved prior to this time point. 6-OHDA is known to undergo rapid auto-oxidation [63], potentially inducing early ROS bursts that were not captured in our experimental design. While sustained oxidative stress was not detected at 120 hpf, the observed increase in apoptosis and tendency toward altered mitochondrial membrane potential suggest mitochondrial involvement in the toxic response under these experimental conditions. However, mitochondrial respiration, ATP production, and electron transport chain activity were not directly assessed in the present study. Thus, our findings indicate mitochondrial participation rather than definitively establishing it as the primary mechanism. Still, mitochondrial membrane integrity is crucial for cellular homeostasis, and its disruption can activate the apoptotic cascade independently of oxidative stress [64]. Although caspase activation was not directly assessed and apoptosis was measured at the whole-larvae level, previous studies have linked 6-OHDA-induced mitochondrial dysfunction to apoptotic signalling pathways [65,66]. Regarding almond skin extract, the lack of ROS modulation under co-exposure conditions was expected given the absence of oxidative stress induced by 6-OHDA alone. Nevertheless, a tendency toward mitochondrial membrane potential stabilization was observed, suggesting a potential role in preserving mitochondrial bioenergetics, although insufficient to fully prevent apoptosis activation. In fact, different natural compounds can modulate and regulate mitochondrial functions [67,68] either by enhancing ATP production, stabilizing mitochondrial dynamics, or influencing the balance between pro- and anti-apoptotic signals. However, further studies are required to elucidate the specific molecular mechanisms involved and determine whether this effect is linked to specific bioactive components present in the extract. Interestingly, when tested alone, the acetonic extract increased GST activity and reduced ROS levels. This effect is likely mediated by its polyphenolic composition [38], as polyphenols are known to enhance endogenous antioxidant defences and reinforce cellular redox homeostasis [69]. By limiting electron leakage and ROS formation, these compounds may indirectly support mitochondrial function. Still, the present study is based on endpoint measurements rather than detailed time-course or pathway-specific analyses. Mitochondrial respiration, ATP production, and caspase activation were not directly assessed. Furthermore, while the zebrafish 6-OHDA model provides valuable in vivo insight, it does not fully recapitulate the complexity of mammalian PD pathology. Future studies incorporating molecular pathway analysis and translational pharmacokinetic evaluation will be necessary to further elucidate the underlying mechanisms.

## 4. Materials and Methods

### 4.1. Reagents and Extract

6-Hydroxydopamine (6-OHDA) hydrobromide (HB1889, CAS 636-00-0, purity > 98%) was purchased from Hello Bio Ltd. (County Meath, Republic of Ireland) and prepared in E3 medium (5 mM NaCl, 0.17 mM KCl, 0.33 mM CaCl_2_, and 0.33 mM MgSO_4_ (pH 7.24) [70]. The solution was stabilized with 0.02% of ascorbic acid to avoid its auto-oxidation [63] and not cause zebrafish developmental changes (up to 0.1%, [46]). The same batch of the acetonic almond skin extract, prepared and chemically characterized before [38] and stocked at a concentration of 40 g/L at −20 °C, was used. All other chemicals were of the highest grade commercially available and obtained from standard commercial suppliers.

### 4.2. Zebrafish Maintenance and Reproduction

Adult AB zebrafish (*Danio rerio*) were housed as previously described [71] in an open water system using 20-L glass tanks, with a density of 2–3 fish per litre [72]. The tanks contained treated tap water from Vila Real that was aerated, dechlorinated, charcoal-filtered, and UV-sterilized. The water quality parameters were regularly monitored and maintained within specific ranges for dissolved oxygen, pH, temperature, conductivity, alkalinity, hardness, and various nitrogen compounds as published elsewhere [30]. The zebrafish were kept on a controlled 10-h dark/14-h light cycle, and their diet consisted of Zebrafeed [73], which was administered twice daily. To obtain embryos, fish pairs were allowed to mate overnight within their tanks. Following spawning, the collected embryos were disinfected with 0.5% chloramine T solution [74]. After disinfection, the embryos were rinsed in E3 medium, and their normal morphology was assessed using an A SMZ 445 stereomicroscope (Nikon, Tokyo, Japan) before use in subsequent experiments.

### 4.3. Experimental Design

**Figure 5:**
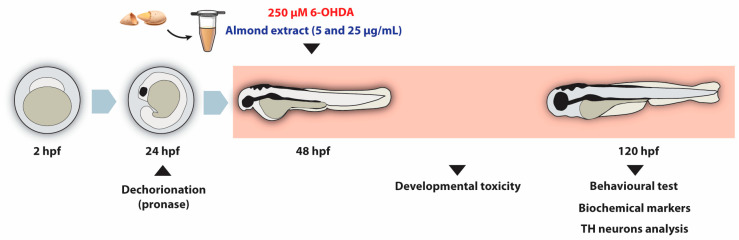
Experimental timeline and analysis overview. Fertilized eggs were collected at approximately 2 h post-fertilization (hpf) and allowed to develop until 24 hpf. At this stage, the chorion was enzymatically removed using pronase. From 48 hpf, larvae were exposed for 72 h to 250 µM 6-OHDA, either alone or in combination with almond extract at concentrations of 5 or 25 µg/mL. Developmental parameters were monitored throughout the experimental period. At the end of the exposure, behavioural assessments, oxidative stress markers, and tyrosine hydroxylase (TH)-positive neurons (via immunofluorescence) were evaluated.

The embryos, at 2 h post-fertilization (hpf), were placed in 6-well plates with 50 randomly selected embryos per well (*n* = 1) in E3 medium. At 24 hpf, the embryos underwent dechorionation using pronase treatment (2 mg/mL, Roche Diagnostics, Mannheim, Germany) [75], followed by two washes with E3 medium. At 48 hpf, the embryos were randomly assigned to different experimental groups: two negative control groups (CTRL: E3 medium and ACE: 0.05% acetone), 250 μM 6-OHDA group, two groups co-treated with 250 μM 6-OHDA and the acetonic almond extract (5 and 25 µg/mL, respectively, E5 and E25), and a group exposed to the highest concentration of the almond extract (25 µg/mL, E25). The 6-OHDA neurotoxin is commonly used to create zebrafish models of PD. At a concentration of 250 μM, it induces dopaminergic neuron death and locomotor deficits [27,28], as well as morphological alterations in embryos [32]. The almond skin extract concentrations were selected according to the toxicological profile previously determined [38]. The experimental protocol was designed to begin exposure to 6-OHDA at 48 hpf, the timing at which all neuronal populations are present [40,76]. The experiment continued until 120 hpf, with solutions being replaced daily to maintain consistent exposure conditions. To ensure reproducibility and statistical power, the experiments were replicated five times using different biological replicates. Throughout the duration of the study, mortality was checked daily, with any dead embryos or larvae promptly removed from the experimental plates to maintain the integrity of the surviving population and prevent potential contamination. The entire experimental procedure, as outlined in Figure 5, strictly adhered to ethical guidelines for animal research (European Directive, 2010/63 and Portuguese Decreto-Lei 113/2013).

### 4.4. Morphological Assessment

The assessment of developmental defects in zebrafish larvae was conducted at three time points: 72, 96, and 120 hpf. The evaluation employed a modified scoring system based on Beekhuijzen et al. [77], which categorized larvae according to the severity of their defects: Class IV: normal development; Class III: one mild malformation; Class II: two malformations; Class I: three or more malformations (Figure 1C). The specific parameters evaluated included cardiac defects (typically manifesting as pericardial edema), head deformities, yolk sac and yolk extension abnormalities, tail irregularities, and eye irregularities. The assessment protocol involved five biological replicates, 10 randomly selected animals from each group per replicate (averaged as *n* = 1), examination of larvae immobilized in 1% methylcellulose inside a glass capillary, and the use of an SMZ800 stereomicroscope (Nikon, Japan) for observation. Additionally, at 120 hpf, the standard body length of the larvae was measured using digital image analysis software (Digimizer version 5.7.2, MedCalc Software Ltd., Ostend, Belgium).

### 4.5. Whole Mount Immunostaining

At 120 hpf, a fluorescent immunohistochemistry technique was applied to visualize tyrosine hydroxylase (TH) positive neurons in the zebrafish larvae. Tyrosine hydroxylase is a key enzyme in the synthesis of dopamine and is commonly used as a marker for dopaminergic neurons [78]. This method was implemented following established protocols previously described by Pinho et al. [79] and Vauti et al. [80]. In brief, ten randomly selected larvae were collected and fixed overnight at 4 °C in 4% paraformaldehyde (PFA) prepared in phosphate-buffered saline (PBS). The samples were then rinsed three times in PBS for 5 min and stored in 100% methanol at −20 °C for a minimum of two days. The larvae were subsequently washed twice in absolute ethanol for 5 min and incubated for 1 h in a 1:1 mixture of ethanol and xylene. Following two washes in absolute ethanol (5 min each), the samples were progressively rehydrated over 15 min using decreasing ethanol concentrations (90%, 75%, 50%, 25%, and 0%) containing 0.1% Tween-20. Permeabilization was performed by immersing the samples in 80% acetone at −20 °C without shaking for 30 min, followed by two washes in PTwx (PBS with 0.1% Triton X-100 and 0.1% Tween-20) for 5 min. The larvae were then bleached for 1 h in a solution of 1.5% hydrogen peroxide (H_2_O_2_) and 50 mM potassium hydroxide (KOH). After permeabilization, the samples were washed in PTwx and incubated overnight at 4 °C in blocking solution (PBS with 1% Triton X-100 and 5% bovine serum albumin, BSA). The following day, the samples were incubated for 16 h in the primary antibody (1:500; MAB318, Merck, Darmstadt, Germany). After five rinses in PTwx, the larvae were incubated overnight with the secondary antibody conjugated to AlexaFluor-488 (1:500; 115-545-003, Jackson ImmunoResearch Europe Ltd., Ely, UK). The samples were then washed three times in PTwx, followed by PBS, and stored in 50% glycerol in PBS. For imaging, the larvae were transferred to 90% glycerol in PBS (pH 8.5–9.0) and visualized in glass capillaries using an Olympus IX51 inverted fluorescence microscope (Nikon, Japan) at 4X magnification. Control larvae were initially used to optimize imaging parameters to ensure non-saturated TH signal detection, and these parameters were maintained constant for all experimental groups. Larvae were positioned consistently to ensure comparable orientation. Regions of interest (ROIs) encompassing the dopaminergic brain region were defined based on anatomical landmarks and applied uniformly to all samples. Mean fluorescence intensity was calculated after background subtraction using identical threshold parameters across blind and coded groups. The average intensity of TH staining in neurons located in the brain region was quantified for each sample (10 larvae considered as *n* = 1, with 5 independent replicates) using ImageJ v1.51 software [81].

### 4.6. Locomotion Activity

At 120 hpf, the locomotor activity of zebrafish larvae was assessed using established protocols [82,83]. This evaluation was conducted in a behavioural room maintained at 27–28 °C and between 09.00 a.m. and 04.00 p.m. using ten undeformed animals (*n* = 1) per each of the 5 replicates to ensure robust and reproducible results. Briefly, the experimental medium was replaced with E3 buffer, and individual larvae were carefully positioned in the centre of a 6-well 0.5% agarose-coated plate. This setup ensured that all experimental groups were subjected to identical environmental conditions during the locomotion assessment. The use of agarose-coated plates helps to minimize reflections from the walls. After a 5 min habituation, the well was filmed from above for 10 min using a mobile phone (1920 × 1080/30 fps) placed above a 15.6″ laptop LCD screen showing a white Microsoft PowerPoint (Microsoft Corp., Washington, DC, USA). The behaviour was then analyzed using the ANY-Maze^®^ v7.08 software (Stoelting CO, Wood Dale, IL, USA), assessing the mean speed, total distance moved, time immobile, mean absolute turn angle, and mean distance to the centre of the well. Upon completion of the locomotor activity analysis, representative tracking images for each experimental group were generated using ANY-Maze^®^ software.

### 4.7. ROS, Apoptosis, and Mitochondrial Membrane Potential (ΔΨm) Measurement

To assess reactive oxygen species (ROS) levels, apoptosis, and mitochondrial membrane potential, additional experimental replicates were conducted under the previously described conditions, following established methods [84]. The assessment of ROS levels involved immersing 10 larvae (*n* = 1) in a dichlorodihydrofluorescein-diacetate solution (DCFH-DA, 20 µg/mL) for 60 min. Mitochondrial membrane potential was evaluated using the fluorescent probe JC-1 (5.0 µM) for 30 min on 10 larvae (*n* = 1) from each replicate. Apoptosis was measured by exposing larvae to acridine orange (AO, 10 µg/mL) for 15 min. Following exposure to each probe, 120 hpf larvae were washed and collected in 200 μL of E3 buffer and stored at −20 °C. Subsequently, the samples were homogenized using a Tissuelyser II (Qiagen, Hilden, Germany) at 30 Hz for 90 s and centrifuged at 12,000× *g* for 10 min at 4 °C. The resulting supernatants were collected, and fluorescence measurements were taken using a Cary Eclipse fluorescence spectrophotometer (Varian, Palo Alto, CA, USA). The excitation/emission wavelengths used were 485/535 nm for ROS, 535/590 nm for mitochondrial membrane potential (ΔΨm), and 485/535 nm for apoptosis.

### 4.8. Biochemical Evaluation

To investigate the biochemical effects of the treatments, ten replicates of 120 hpf larvae from each experimental group were processed as previously described [85,86]. The larvae were homogenized in 400 μL of cold buffer (0.32 mM sucrose, 20 mM HEPES, 1 mM MgCl_2_, and 0.5 mM phenylmethyl sulfonylfluoride (PMSF), pH 7.4). The homogenate was then centrifuged at 12,000× *g* for 10 min at 4 °C, and the resulting supernatant was used for various biochemical analyses performed at 30 °C. The activities of several antioxidant enzymes were measured spectrophotometrically: superoxide dismutase (Cu/Zn-SOD) at 560 nm, catalase (CAT) at 240 nm, glutathione peroxidase (GPx), and glutathione reductase (GR) both at 340 nm. These assays involved measuring the reduction of nitroblue tetrazolium (NBT) for Cu/Zn-SOD, H2O2 decomposition for CAT, and NADPH disappearance for GPx and GR. Glutathione-s-transferase (GST) activity was determined by monitoring the production of glutathione-dinitrobenzene conjugate at 340 nm. Glutathione levels, both reduced (GSH) and oxidized (GSSG), were measured at 320 nm excitation and 420 nm emission wavelengths, which were used to calculate the glutathione redox ratio (oxidative stress index, OSI). Lipid peroxidation (LPO) was assessed by measuring malondialdehyde-thiobarbituric acid adducts at 530 nm, with a correction for non-specific adducts at 600 nm. The DNA strand breaks were quantified at 360 nm for excitation and 450 nm for emission by the method previously described [87]. Total protein concentration was estimated at 280 nm using bovine serum albumin (BSA) as a standard [88]. All spectrophotometric measurements were conducted using a PowerWave XS2 microplate scanning spectrophotometer (BioTek Instruments, Winooski, VT, USA), while fluorescence measurements were performed on a Cary Eclipse fluorescence spectrophotometer (Varian, Palo Alto, CA, USA).

### 4.9. Statistical Analysis

The statistical analysis was conducted using a systematic approach to ensure appropriate treatment of the results. Initially, the Shapiro–Wilk test was used to assess the normality of data distribution, followed by the Brown-Forsythe test to evaluate the equality of population variances. For data that met the assumptions of normality, one-way analysis of variance (ANOVA) was performed, followed by Tukey’s post-hoc multiple comparison test to identify specific group differences. Results from these analyses were presented as mean values with standard deviations. In cases where data did not conform to a normal distribution, the non-parametric Kruskal–Wallis test was applied, followed by Dunn’s pairwise comparison test for post-hoc analysis. Results from these non-parametric tests were reported as median values with interquartile ranges. To maintain data integrity, Grubb’s outlier test was utilized to identify and remove any outliers from the dataset. Throughout the analysis, a *p*-value less than 0.05 was considered statistically significant. All statistical analyses and graphical representations were performed using GraphPad Prism 9.1 software (GraphPad Software, La Jolla, CA, USA).

## 5. Conclusions

In summary, this study suggests that the almond skin acetonic extract modulates dopaminergic and behavioural alterations in a zebrafish model of PD. While 6-OHDA exposure caused reduced TH immunoreactivity, behavioural impairments, and increased apoptosis, co-exposure to the extract mitigated neuronal damage and restored growth parameters. Behavioural outcomes were dose-dependent, highlighting the complexity of plant-derived regulatory mechanisms. Although overt oxidative stress was not detected at 120 hpf, the observed mitochondrial membrane potential alterations and apoptosis suggest mitochondrial involvement in 6-OHDA-induced toxicity under these experimental conditions. Further mechanistic studies are required to clarify the temporal sequence and causal hierarchy of these events. Notwithstanding, these findings position almond skin as a promising source of neuroprotective bioactive compounds, although further studies addressing bioavailability, pharmacokinetics, and brain penetration are required to fully assess its therapeutic relevance for neurodegenerative disorders, such as PD.

## Figures and Tables

**Table 1:** Biochemical profile after exposure to 6-OHDA, the almond extract, and its combinations, in zebrafish larvae.

	Experimental Groups						Statistical Test	*p*-Value
Parameter	CTRL	ACE	6-OHDA	6-OHDA+E5	6-OHDA+E25	E25		
SOD	**58.6 (20.8–90.5) ^ab^**	**118.0 (77.8–179.3) ^a^**	**71.5 (57.8–93.1) ^ab^**	**68.8 (44.5–87.1) ^ab^**	**68.1 (53.9–120.6) ^ab^**	**41.4 (28.9–50.4) ^b^**	**X^2^(5) = 11.30**	**0.046**
CAT	**148.6 (95.9–189.2) ^ab^**	**209.3 (171.9–252.3) ^ab^**	**86.7 (52.8–155.1) ^ab^**	**42.9 (29.2–110.9) ^b^**	**262.7 (230.4–296.2) ^a^**	**256.9 (196.2–299.4) ^a^**	**X^2^(5) = 21.77**	**0.0006**
GPx	49.0 ± 21.0	56.8 ± 19.4	47.9 ± 9.2	45.1 ± 22.2	44.5 ± 14.2	56.3 ± 16.2	F(5, 24) = 0.446	0.812
GR	7.7 (7.1–16.7)	7.0 (4.1–7.7)	8.3 (4.4–11.9)	8.6 (5.8–13.8)	6.7 (2.8–13.5)	5.9 (3.5–10.2)	X^2^(5) = 3.797	0.579
GST	**8.0 (3.8–15.2) ^b^**	**26.3 (20.2–29.8) ^ab^**	**16.8 (15.8–20.8) ^ab^**	**13.0 (9.7–25.8) ^ab^**	**17.2 (9.4–16.4) ^ab^**	**27.8 (25.1–33.8) ^a^**	**X^2^(5) = 14.53**	**0.013**
GSH	18.1 ± 2.8	31.6 ± 11.4	20.7 ± 4.3	22.7 ± 6.9	21.1 ± 9.2	22.7 ± 4.0	F(5, 24) = 2.095	0.101
GSSG	87.7 ± 10.6	103.1 ± 16.1	86.9 ± 21.7	85.3 ± 4.4	71.5 ± 22.1	85.3 ± 10.1	F(5, 24) = 2.093	0.101
OSI	0.2 ± 0.0	0.3 ± 0.1	0.3 ± 0.1	0.3 ± 0.1	0.4 ± 0.3	0.3 ± 0.1	F(5, 24) = 0.785	0.571
LPO	8.9 (5.8–9.7)	5.6 (4.8–6.2)	6.4 (5.4–8.9)	5.5 (4.2–7.0)	4.8 (3.1–5.1)	8.1 (5.1–10.1)	X^2^(5) = 10.16	0.071
PC	1.7 ± 1.2	3.3 ± 1.6	2.3 ± 0.4	2.8 ± 0.7	3.2 ± 1.8	2.6 ± 0.7	F(5, 24) = 1.277	0.306
DNA	69.2 ± 41.1	59.6 ± 36.9	68.1 ± 18.8	42.9 ± 16.6	65.2 ± 24.6	61.4 ± 47.6	F(5, 24) = 0.424	0.827

Superoxide dismutase (SOD) and catalase (CAT) activity are expressed as U/mg protein. Glutathione Peroxidase (GPx) and Glutathione Reductase (GR) activities are expressed as nmol NADPH/min.mg protein. Glutathione-S-Transferase (GST) activity is expressed as nmol CDNB/min.mg protein. Oxidized (GSSG) and reduced (GSH) glutathione levels are expressed as μmol GSH and μmol GSSG, respectively, per mg of protein. The Oxidative Stress Index (OSI) is the quotient between GSH and GSSG levels. Lipid peroxidation (LPO) is shown as μmol MDA/mg protein. Protein carbonylation (PC) is shown as nmol DNPH/mg protein, whereas DNA damage is quantified as µg DNAsb/mg protein. Data from independent replicates are expressed as mean ± SD for parametric data distribution or median (25th–75th quartile) for non-parametric data. Statistical analysis was performed using one-way ANOVA followed by Tukey’s multiple-comparison test or Kruskal–Wallis followed by Dunn’s test. Statistical differences are highlighted in bold and different lowercase letters indicate significant differences between groups (*p* < 0.05). CTRL (E3 medium), ACE (0.05% acetone), 6-OHDA (250 µM), 6-OHDA+E5 (250 µM 6-OHDA + 5 µg/mL extract), 6-OHDA+E25 (250 µM 6-OHDA + 25 µg/mL extract), and E25 (25 µg/mL extract).

## Data Availability

The dataset is available on request from the authors.
